# Factors associated with functional loss among community-dwelling Mexican older adults

**DOI:** 10.7705/biomedica.5380

**Published:** 2020-06-30

**Authors:** Nicolás Castellanos-Perilla, Miguel Germán Borda, Álvaro Fernández-Quilez, Vera Aarsland, Hogne Soennesyn, Carlos Alberto Cano-Gutiérrez

**Affiliations:** 1 Semillero de Neurociencias y Envejecimiento, Instituto de Envejecimiento, Facultad de Medicina, Pontificia Universidad Javeriana, Bogotá, D.C., Colombia Pontificia Universidad Javeriana Instituto de Envejecimiento Pontificia Universidad Javeriana BogotáD.C Colombia; 2 Centre for Age-Related Medicine (SESAM), Stavanger University Hospital, Stavanger, Norway Stavanger University Hospital Stavanger Norway; 3 Faculty of Health Sciences, University of Stavanger, Stavanger, Norway University of Stavanger Stavanger Norway; 4 Stavanger Medical Imaging Laboratory, Stavanger University Hospital, Stavanger, Norway Stavanger University Hospital Stavanger Norway; 5 Faculty of Science and Technology, University of Stavanger, Stavanger, Norway University of Stavanger Stavanger Norway; 6 School of Medicine, Semmelweis University, Budapest, Hungary Semmelweis University Budapest Hungary; 7 Unidad de Geriatría, Hospital Universitario San Ignacio, Bogotá, D.C., Colombia Hospital Universitario San Ignacio BogotáD.C Colombia; ‡ Shared first authorship Shared first authorship

**Keywords:** Elderly, public health, pain, activities of daily living, anciano, salud pública, dolor, actividades cotidianas

## Abstract

**Introduction:**

Functional status decline is related to many negative outcomes.

**Objective:**

To explore the relationship of sociodemographic, medical, and psychological factors with the incidence of functional status decline in Mexican older adults.

**Materials and methods:**

Data from the 2012 and 2015 waves of the Mexican Health and Aging Study (MHAS) survey were analyzed. Participants with previous functional status decline at baseline were excluded. We assessed functional status decline individually with activities of daily living (ADL) and instrumental ADL (IADLs) in an individual way.

**Results:**

Age was associated with functional limitations in ADL. Being male had an association with limitations for IADL. A poor financial situation and lower education related to higher limitations for ADL. Furthermore, pain, comorbidities, and depression were found to be independently associated with limitations in ADL. IADL limitation was associated with age, poor education, comorbidities, and depression, as well as cognitive impairment.

**Conclusions:**

We found that factors such as age, financial status, educational level, pain, and the number of comorbidities were associated with the incidence of functional status decline. Pain had a greater association in the 3-year functional ADL decline incidence when compared with cognitive impairment. Studying functional decline by domains allowed us to find more detailed information to identify factors susceptible to intervention with the aim to reduce the incidence of functional status decline and dependence.

## Resumen

**Introducción.:**

El deterioro funcional está relacionado con muchos resultados adversos.

**Objetivo.:**

Explorar la relación de los factores sociodemográficos, médicos y psicológicos con la incidencia del deterioro funcional en los adultos mayores mexicanos.

**Materiales y métodos.:**

Se analizaron los datos de las cohortes de 2012 y 2015 de la encuesta del Estudio Mexicano de Salud y Envejecimiento. Se excluyeron los participantes con discapacidad funcional en el período de referencia (2012). Se evaluó de forma individual el deterioro funcional en las actividades básicas de la vida diaria (AVD) y en las instrumentales (AIVD).

**Resultados.:**

Se encontró que el dolor, las comorbilidades, el nivel educativo, el estatus socioeconómico y la depresión se asociaban independientemente con el deterioro de las AVD. El deterioro de las AIVD se asoció con la edad, la educación deficiente, las comorbilidades, la depresión y el deterioro cognitivo.

**Conclusiones.:**

La edad, el sexo, el estado financiero, el nivel educativo, el dolor y el número de comorbilidades se asociaron con la incidencia del deterioro funcional. El dolor tuvo una mayor asociación con la incidencia del deterioro funcional en las AVD a los tres años, en comparación con el deterioro cognitivo. El estudio del deterioro funcional por dominios permitió recabar información más detallada para determinar los factores que pueden intervenirse con el objetivo de reducir la incidencia del deterioro funcional y la dependencia.

The number of people over 60 years worldwide is expected to double by 2050. In Latin America, this growth is projected to be up to 80% and it is expected that by this year, 21% of the population will be 60 years or older and rise to almost 36% by 2100; these populations are also aging at a faster rate than North American and European populations ^1^^-^^4^. However, this growth comes with challenges, such as the increase of geriatric syndromes, chronic diseases, and sensory deficits, which have repercussions in terms of independence and functionality ^3^.

Functionality can be defined as the ability of individuals to meet their needs autonomously, independently, and satisfactorily ^5^. Activities of daily living (ADL), divided into basic ADL and instrumental ADL (IADL), have been broadly used as a measure of functional status ^6^^,^^7^. ADL include activities for basic functioning, self-care mobility, and survival, whereas IADL include activities that are necessary to live in a community such as managing money, cooking, or shopping for groceries ^8^. Facing issues when performing such activities is closely related to frailty and disability which leads to frequent hospitalizations, nursing home admission, depression, morbidity, and death ^9^. The decline in just one of the ADL has detrimental effects on the quality of life and generates a large degree of dependence, which is currently a public health issue due to its negative consequences and constantly growing prevalence ^10^^,^^11^.

The functional status decline among adults aged 45 and older is higher in low-income countries compared to high-income countries ^10^. Moreover, people with limitations in ADL and disability experience worse labor market outcomes, have a higher likelihood of being poor and of having a lower educational level than those without functional disability. A decline in functional status increases health care costs due to the need for a permanent caregiver, specialized health care, and institutionalization ^12^^,^^13^. Additionally, subjects with limitations in ADL face a number of obstacles including environmental and institutional barriers, which prevent their full and equal participation in all aspects of life. Older people with functional limitations are among the most adversely affected, as they have to face even greater barriers in society ^11^.

The importance of evaluating the ability to perform activities of daily living in the older age group has been widely emphasized in geriatric medicine and, consequently, several instruments to assess functionality are available ^14^. Functional status depends on multiple non-modifiable factors such as sex and age. Nevertheless, many factors contributing to functional status decline are preventable or modifiable, which should be taken into consideration in the design of public health policies ^15^.

According to the World Health Organization, 80% of people with disabilities live in low-income nations like Latin American countries. The SABE survey showed there is a higher prevalence of comorbidities and limitations in ADL in the region. Furthermore, associations between the functional status decline measured with limitations in ADL and IADL and the number of comorbidities and sociodemographic factors (age, being female, fewer years of schooling) have been reported ^4^^,^^16^. Other factors related to functional status decline include socioeconomic status, which was found to be higher among those with a lower socioeconomic condition in a Chilean cohort ^17^. The higher incidence and prevalence of obesity is correlated with cardiovascular disease and does affect functional status in the region ^4^.

Given the importance of understanding functional decline, our aim in the present study was to determine factors that may be associated with the incidence of functional status decline in a longitudinal three-year analysis of community-dwelling Mexican older adults.

## Materials and methods

### Sample

We conducted a secondary analysis of the Mexican Health and Aging Study (MHAS). The MHAS is a longitudinal study in five waves designed using probabilistic sampling to obtain a national representative sample of the Mexican population aged 50 years or older ^18^. The study includes data regarding socio-demographic aspects, health-related issues, accessibility to health services, cognitive performance, functional status, and financial resources.

**Figure 1:**
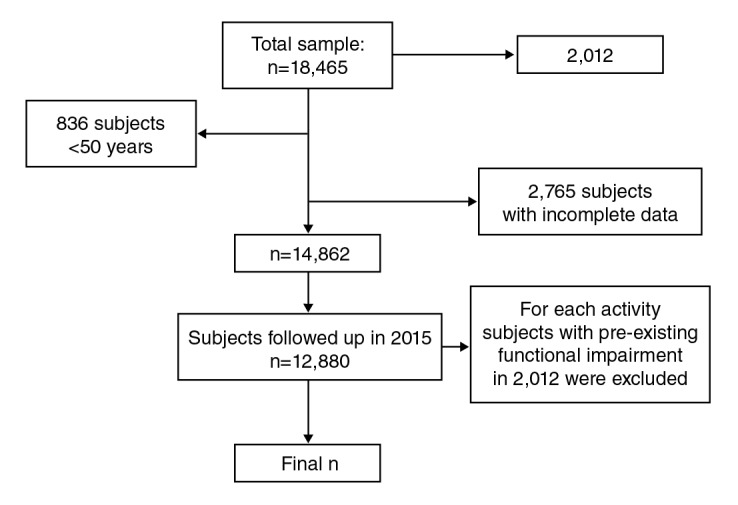
Sample selection

The two waves conducted in 2012 and 2015 were analyzed to assess the three-year functional decline incidence. The total sample used for the current study consisted of 12,880 subjects. For each activity, subjects with preexisting functional impairment in 2012 were excluded. The criteria for the selection of the subjects are shown in figure 1.

n=number of participants

### Measurements

*Dependent variable.* We used a translated version of the Katz index of activities of daily living ^19^^,^^20^. The variable was dichotomized defining functional status decline as having difficulties or receiving help to perform activities of daily living or instrumental ADL. ADL included activities such as dressing, bathing, eating, getting in and out of bed, or toileting. IADL were assessed by the Lawton Brody scale ^20^^,^^21^ and included preparing meals, taking medications, shopping for groceries or clothes, and managing money. All the activities were dichotomized assigning a value of 0 if help was not required to perform the activity and 1 (disabled) if any help was needed or participants answered “does not do” or “cannot do”, as done in previous studies ^22^.

*Independent variables.* Sociodemographic variables included age, educational level, financial status, and marital status. Age and educational level were analyzed in years as continuous variables. The financial status was self-reported and dichotomized in a way such that subjects reporting an “excellent”, “very good” or “good” financial status were considered to have a good financial situation and subjects answering “fair” or “poor” were categorized as having a poor one. Marital status was also dichotomized as not in a relationship or in a relationship (single, divorced, separated, or widowed were considered as not in a relationship whereas married or any other union was considered to be in a relationship).

*Other independent variables.* The cognitive function was evaluated using the Cross-Cultural Cognitive Examination test (CCCE). The CCCE has a maximum score of 80 points and includes the evaluation of several cognitive domains such as primary verbal memory, selective attention, secondary verbal memory, executive function and motor control, and visual memory ^23^. We used a translated version of this test ^24^. Cut-off points for cognitive impairment were set using the 10^th^ percentile by sex and educational level. We dichotomized the variable using the aforementioned cut-off ^25^.

Comorbidities were considered as a single continuous variable. The variable was created based on the sum of the six most frequent comorbidities (arthrosis, respiratory issues, previous heart attack or infarction, diabetes, hypertension, and stroke). The data on comorbidities were obtained through self-report.

As for depression, the MHAS screening questionnaire was used to assess depressive symptoms including nine questions with yes/no answers: Within the past week, was the respondent ^1^ Depressed?, ^2^ Experiencing difficulty performing?, ^3^ Experiencing restless sleep?, ^4^ Happy?, ^5^ Lonely?, ^6^ Enjoying life?, ^7^ Sad?, ^8^ Feeling tired?, ^9^ Energetic? The cut-off value for depressive symptoms was defined as a score of ≥5 points ^26^. Finally, we also dichotomized the variable categorizing those with ≥5 points as having depressive symptoms.

### Statistical analysis

Results from the descriptive analyses were expressed as frequencies and percentages for categorical variables while standard deviation (SD) and means were used for continuous variables. Then, we performed a bivariate comparative analysis of the differences in the incidence of functional impairment in ADL. The chi-squared and t tests were used where appropriate. Finally, we did a multivariate analysis using problems in ADL and IADL as dependent variables and adjusting for confounding factors such as age, sex, financial situation, marital status, education, comorbidities, depression, cognitive impairment, number of medical visits, and presence of pain. Next, we obtained odds ratios (OR) with 95% confidence intervals (CI) where p values lower than 0.05 (p<0.05) were considered statistically significant. The data were analyzed using Stata 14^™^ for Mac OS.

### Ethical issues

This study is a secondary analysis of the MHAS study approved by the *Pontificia Universidad Javeriana*, Bogotá, Colombia. MHAS approval was sought and obtained from the Institutional Review Board of the University of Texas Medical Branch, and the Mexican *Instituto Nacional de Estadística y Geografía* and *Instituto Nacional de Salud Pública.* All study subjects signed an informed consent. The study followed the ethical guidelines of the Declaration of Helsinki.

## Results

**Figure 2:**
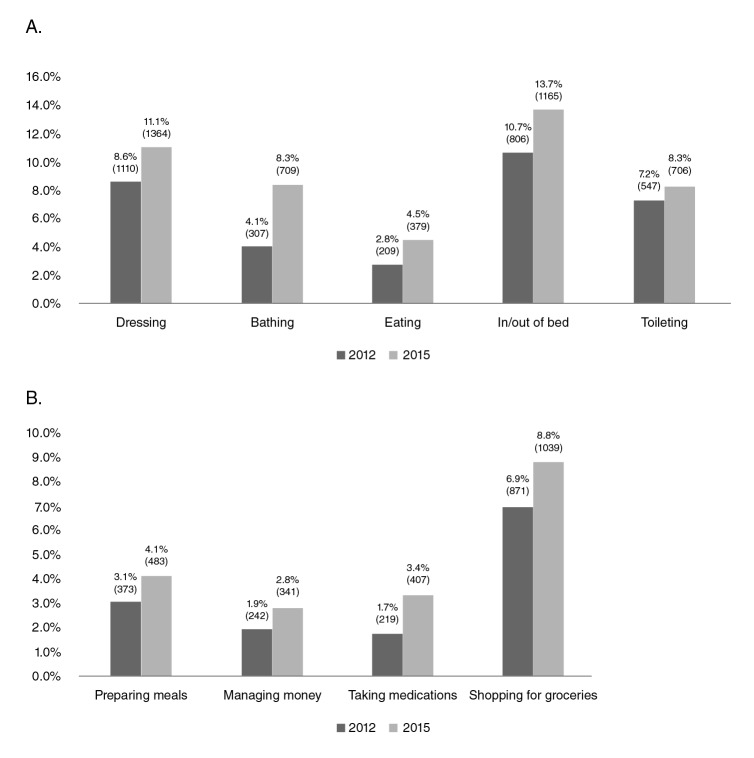
A. ADL and B. IADL disability transition between 2012 and 2015

The cohort baseline details are provided in table 1. The prevalence of functional limitation by activity is detailed in figure 2. The mean age of the sample was 63.88 ± 9.21 years and the majority of subjects were female (56.01%). Figure 2 shows the transition of ADL and IADL limitations between 2012 and 2015: getting in/out of bed (ADL) had the highest incidence (3%) while taking medications was the IADL with the highest incidence (1.7%).

**Table 1:** Sample description

Sociodemographics		
Age (mean, SD)	63.88	(9.21)
Gender		
Male	56.66	(43.99)
Female	72.14	(56.01)
Financial situation		
Good	28.41	(22.08)
Poor	100.28	(77.92)
Marital status		
Single	9.65	(18.51)
In a relationship	42.48	(81.49)
Education (years)	7.32	(5.73)
Baseline comorbidities	0.86	(0.89)
Pain	48.59	(37.75)
Medical visits last year		
None	3.501	(27.18)
One or more	93.79	(72.82)
Baseline cognitive limitation	4.58	(6.75)
Baseline pain	48.59	(37.75)
Baseline depres	43.96	(34.13)
Baseline IADL limitation		
Preparing meals	3.73	(3.05)
Managing money	2.42	(1.89)
Taking medications	2.19	(1.72)
Shopping for groceries	8.71	(6.93)
Baseline ADL limitation		
Dressing	11.10	(8.62)
Bathing	3.07	(4.06)
Eating	2.09	(2.77)
Transferring in and out of bed	8.06	(10.65)
Toileting	5.47	(7.24)

IADL: Instrumental activities of daily living (mean, SD) or (n, %); ADL: Activities of daily living

In the bivariate analysis, statistically significant differences were found in the incidence of functional status decline evaluated by ADL and IADL (table 2). Finally, we performed a multivariate analysis (table 3) to evaluate the association between the factors explored in 2012 and the development of limitations for ADL and IADL three years later. Several factors were found to be independently associated with functional limitations for IADL. Age was associated with the inability to prepare meals (OR=1.08; 95% CI: 1.071.10; p=0.023) and managing money (OR=1.04; 95% CI: 1.01-1.01; p=0.016). Higher education was inversely associated with the inability to prepare meals (OR=0.80; 95% CI: 0.68-0.94; p=0.008), taking medications (OR=0.83; 95% CI: 0.72-0.97; p=0.021), and managing money (OR=0.93; 95% CI: 0.87-0.99; p= 0.036). Comorbidities (OR=1.62; 95% CI: 1.21-2.17; p<0.001) and depression (OR=2.51; 95% CI: 1.40-4.49; p=0.002) were associated with functional loss in managing money. Finally, cognitive impairment was associated with disability in preparing meals (OR=3.94; 95% CI: 1.01-15.30; p=0.047).

**Table 2:** Bivariate analysis for instrumental activities of daily living limitations incidence

IADL	n (%) p or mean (SD) 95% IC p			
Variable	Preparing meals	Managing money	Taking medications	Shopping for groceries
Sociodemographics				
Age	69.81 (9.64) <0.001	71.38 (10.26) <0.001	71.59 (9.83) <0.001	68.33 (9.58) <0.001
Gender				
Male	106 (2.39), <0.001	95 (1.79), <0.001	130 (2.51) 0.01	202 (4.09), <0.001
Female	238 (3.65)	194 (2.90), <0.001	218 (3.28) 0.01	500 (8.32), <0.001
Financial situation				
Good	42 (1.70) <0.001	39 (1.46) <0.001	53 (2.02) 0.001	91 (3.62) <0.001
Poor	302 (3.56)	249 (2.67)	295 (3.21) 0.001	609 (7.23) <0.001
Marital status				
Single	287 (3.97) <0.001	241 (3.02) <0.001	306 (3.89) <0.001	581 (8.22) <0.001
In a relationship	57 (1.52)	48 (1.10)	42 (1.06)	121 (3.12) 0.278
Education	5.47 (3.99) 0.004	5.26 (5.23) 0.006	6.98 (13.22) 0.61	5.68 (4.51), 4.97-6.40, <0.001
Pain	193 (4.79) <0.001	147 (3.30) <0.001	181 (4.07) <0.001	395 (10.23) <0.001
Comorbidities	1.21 (0.88) <0.001	1.17 (0.97) <0.001	1.28 (1.01) <0.001	1.24 (1.01) <0.001
Depression	171 (4.66) <0.001	153 (3.81) <0.001	172 (4.32) <0.001	335 (9.63) <0.001
Cognitive impairment	23 (6.53) <0.001	14 (3.54) 0.156	25 (6.35) <0.001	40 (11.56) 0.005
Medical visits last year				
None	76 (2.51) 0.020	63 (1.91) 0.030	58 (1.84) <0.001	139 (4.47) <0.001
One or more	268 (3.38)	226 (2.60)	290 (3.34)	563 (7.18)

IADL: Instrumental activities of daily living for which help is required or subjects were unable to perform.

**Table:** 

ADL	n (%) p or mean (SD) CI95% p				
Variable	Dressing	Bathing	Eating	Getting into/out of bed	Toileting
Sociodemographics					
Age	67.49 (9.53) <0.001	72.99 (9.67) 72.12- 73.85. <0.001	72.34 (10.19) 71.15-73.55. <0.001	69.11 (9.81) 68.40-69.84. <0.001	69.78 (9.83) 68.90-70.66. <0.001
Gender					
Male	395 (40.72) <0.001	164 (8.53). 0.84	102 (5.32) 0.018	223 (12.30). 0.09	156 (8.36). 0.526
Female	575 (59.28)	318 (8.37)	176 (4.52)	487 (13.97)	323 (8.87)
Financial situation					
Good	130 (13.40) <0.001	71 (7.43) 0.228	43 (4.45) 0.593	94 (10.28) 0.002	53 (5.76) 0.001
Poor	840 (86.60)	410 (8.61) 0.228	235 (4.85) 0.593	616 (14.07)	426 (9.30)
Marital status					
Alone	289 (40.48) <0.001	433 (9.64) <0.001	239 (5.21) 0.003	602 (14.44) <0.001	410 (9.52) <0.001
In a relationship	425 (59.52)	49 (3.98)	39 (3.18)	108 (9.57) 0	69 (5.74)
Education	5.51 (3.99) <0.001	4.37 (3.67) 3.42-5.33. 0.01	4.63 (3.04) 3.50-5.77. 0.05	5.20 (3.86) 4.56-5.84. 0.02	5.51 (4.13) 0.17
Pain	524 (54.02) <0.001	270 (8.76) 0.28	152(4.85) 0.75	451 (16.56) <0.001	290 (9.96) <0.001
Comorbidities	1.94 (0.88) <0.001	1.34 (1.01) <0.001	1.36 (0.99) <0.001	1.37 (0.99) <0.001	1.41 (1.01) <0.001
Depression	448 (46.19) <0.001	244 (9.34) 0.022	146 (5.45) 0.028	388 (16.65) <0.001	2151 (10.14) <0.001
Cognitive impairment	40 (6.69) 0.805	32 (13.06) 0.007	15 (5.88) 0.303	38 (16.45) 0.154	20 (8.66) 0.823
Medical visits last year					
None	130 (35.04) <0.001	89 (8.07) 0.221	42 (3.84) 0.104	117 (11.07) 0.013	67 (6.31) 0.002
One or more	241 (64.96)	393 (8.51)	236 (5.00)	593 (13.98)	412 (9.27)

IADL: Instrumental activities of daily living for which help is required or subjects were unable to perform

**Table 3:** Multivariate analysis for instrumental activities of daily living and activities of daily living limitations incidence

ADL	n (%) p or mean (SD) 95%CI p			
Variable	Preparing meals	Managing money	Taking medications	Shopping for groceries
Age	1.08 (1.07-1.10) 0.023	1.04 (1.01-1.01) 0.016	1.05 (0.98-1.12) 0.146	0.99 (0.89-1.12) 0.991
Gender (male)	1.67 (0.49-5.71) 0.412	1.00 (0.56-1.79) 0.999	3.87 (0.87-17.30) 0.076	2.19 (0.31-15.49) 0.432
Good financial situation	1.33 (0.35-5.01) 0.676	0.94 (0.45-1.98) 0.876	1.53 (0.41-5.78) 0.526	0.22 (0.02-1.70) 0.146
Marital status (Single)	-	0.71 (0.33-1.50) 0.374	4.43 (0.99-19.76) 0.051	1.22 (0.17-1.70) 0.146
Pain	1.83 (0.62-5.42) 0.27	0.58 (0.32-1.06) 0.078	1.09 (0.35-3.39) 0.872	0.51 (0.16-1.65) 0.263
Education	0.80 (0.68-0.94) 0.008	0.93 (0.87-0.99) 0.036	0.83 (0.72-0.97) 0.021	0.98 (0.88-1.09) 0.768
Comorbidities	1.21 (0.66-2.18) 0.53	1.62 (1.21-2.17) <0.001	1.12 (0.61-2.04) 0.720	0.93 (0.49-1.77) 0.837
Depression	1.41 (0.46-4.30) 0.54	2.51 (1.40-4.49) 0.002	1.97 (0.63-6.14) 0.241	1.18 (0.39-3.58) 0.768

IADL: Instrumental activities of daily living for which help is required or subjects were unable to perform

**Table:** 

ADL	n (%) p or mean (SD) 95%CI p				
Variable	Dressing	Bathing	Eating	Getting into/out of bed	Toileting
Age	1.05 (1.02-1.08) <0.001	1.07 (1.01-1.16) 0.037	1.05 (1.01-1.10) 0.014	1.01 (0.98-1.04) 0.414	1.04 (1.03-1.05) <0.001
Gender (male)	1.35 (0.90-2.01) 0.136		1.54 (0.82-2.90) 0.178		
Good financial situation	1.06 (0.67-1.70) 0.783			0.54 (0.28-1.03) 0.062	0.64 (0.48-0.87) 0.005
Marital status (Single)	1.04 (0.68-1.60) 0.844	0.53 (0.14-2.02) 0.360	0.98 (0.82-2.90) 0.178	1.02 (0.67-1.54) 0.933	0.96 (0.70-1.30) 0.807
Pain	2.28 (1.53-3.40) <0.001			1.48 (1.01-2.16) 0.041	1.30 (1.06-1.58) 0.011
Education	0.98 (0.94-1.02) 0.382	0.94 (0.83-1.07) 0.254	0.93 (0.86-1.00) 0.063	0.96 (0.91-1.00) 0.062	
Comorbidities	1.30 (1.07-1.57) 0.08	1.13 (0.71-1.80) 0.617	1.12 (0.83-1.53) 0.451	1.16 (0.95-1.41) 0.134	1.33 (1.20-1.47) <0.001
Depression	1.49 (1.05-2.12) 0.025	2.67 (0.98-7.28) 0.054	1.45 (0.80-2.65) 0.220	1.61 (1.11-2.34) 0.012	1.23 (1.01-1.50) 0.043
Cognitive impairment		1.78 (0.47-6.71) 0.392			
Medical visits last year	1.38 (0.77-2.47) 0.285			1.38 (0.86-2.19) 0.176	1.26 (0.96-1.66) 0.096

ADL: Basic activities of daily living for which help is required or subjects were unable to perform

As for ADL, pain was associated with problems for dressing (OR=1.05; 95% CI: 1.02-1.08; p<0.01), getting in and out of bed (OR=1.48; 95% CI: 1.012.16; p=0.041), and toileting (OR=1.30; 95% CI; 1.06-1.58; p=0.011). Age was associated with limitations in dressing (OR=1.05; 95% CI: 1.03-1.08; p<0.001), bathing (OR=1.07; 95% CI: 1.01-1.16; p=0.037), eating (OR=1.05; 95% CI: 1.01-1.10; p=0.014), and toileting (OR=1.04; 95% CI: 1.03-1.05; p<0.001). A good financial situation was related to a lower chance of toileting limitations (OR=0.64; 95% CI: 0.48-0.87; p=0.005). In addition, comorbidities were associated with a functional decline in dressing (OR=1,30; 95% CI: 1.07-1.57; p=0.008), and in toileting (OR=1.33; 95% CI: 1.20-1.47; p<0.001). Likewise, depression was associated with difficulties getting in and out of bed (OR=1.61; 95% CI: 1.11-2.34; p=0.012) and toileting (OR=1.23; 95% CI: 1.01-1.50; p=0.043).

## Discussion

The significant factors associated with ADL disability were pain, age, poor finances, comorbidities, depression, and frequent medical visits whereas age, poor education, depression, comorbidities, and cognitive impairment were identified as risk factors for IADL disability.

We report that pain significantly affected the ADL, specifically dressing, getting in/out of bed, and using the toilet, in other words, ADL that require more range of movement. Additionally, cognitive decline was only associated with difficulties in preparing meals, which is a complex activity. However, cognitive impairment did not affect ADL the same way pain did suggesting that pain may have a very important and faster impact on functional status decline, which manifests in the individual's basic daily functioning even before cognitive impairment.

Regarding socio-demographic factors, female gender, age, financial situation, and lower education were found to be risk factors for the development of functional status decline. These have been addressed by previous studies showing similar significant associations with the total functional capacity ^6^^,^^16^^,^^27^^-^^32^. Nevertheless, we did not find significant associations between sex and functional status decline.

Studies in other populations have identified the presence of pain as an important risk factor for functional decline. The risk of disability increases in subjects with daily pain and with the pain severity level ^33^. Additionally, pain reduces the range of joint movement, which also limits daily functioning ^34^^-^^36^. Therefore, it is relevant to assess and manage pain in older adults with the aim of promoting physical functioning.

Previous studies in Latin American populations have reported the impact of a poor socioeconomic status and limitations in ADL shown by toileting limitation in our results. Older age, lower education, and the number of comorbidities have been reported to be important factors for functional status decline ^16^, which we found to impact both ADL and IADL ^17^. Moreover, female gender has been found to be related to fewer limitations for IADL, which is probably due to the social role of women in household chores ^27^. However, we did not find statistically significant associations. Previous studies have also mentioned the impact of socioeconomic conditions on functional status decline ^17^, which is similar to our findings for one of the ADL.

Previous reports using MHAS data from the first three waves (2001 to 2012) showed that 42.8% of participants with no limitations in 2001 remained free from limitations in 2012 ^37^^,^^38^. Furthermore, they also remained free of associations of functional decline with age, female gender, and having depression. These studies analyzed the functional decline in terms of basic and instrumental activities. Nonetheless, our study provides more information regarding each activity individually and a larger number of factors. Additionally, we found associations between some of the factors that were not included in previous studies (such as pain and years of education) and functional decline.

Studying functionality from a domain point of view is relevant since impairment in any of the ADL or IADL generates a certain degree of dependence and it is something that functional scales do not show at a first glance. This more detailed view allowed us to identify conditions and factors that are usually under-assessed, for example, pain ^37^.

Our study has some limitations. First of all, it is based on selfreports, which allows for potential memory bias. Moreover, the number of comorbidities that were taken into account was limited. The severity of pain was not assessed and the association between pain and functional decline could be derived from an underlying disease not directly addressed. Sociodemographic factors like education are related to health access in communities and, as such, to the treatment and prevention of disability. Moreover, this is a secondary analysis of a study that was not specifically designed to resolve our hypothesis.

On the other hand, our study has several strengths: it is based on one of the largest surveys available in Latin America aimed at the study of older adults. Also, we used data from a country-representative sample of older adults in México. Furthermore, our results are consistent with the existing literature. Finally, this is one of the few studies exploring the association of different factors with functional decline in Latin America.

The functional status decline was associated with factors such as age, educational level, comorbidities, depression, pain, female gender, and having a poor financial situation. When comparing pain with cognitive impairment, the first one had a greater influence in the three-year incidence of functional basic ADL decline.

Some of the significant risk factors of functional impairment can be potentially modified or prevented and, therefore, they can be used as targets for preventing disability in the old age. For instance, socioeconomic disparities have an impact on functional status, so reducing inequalities can prevent disability in the older age ^4^. Low education, a high number of comorbidities, and common risk factors such as obesity and a high prevalence of cardiovascular diseases in these populations could be other starting points for interventions aiming to prevent functional status decline ^4^^,^^16^. Our findings show that functional decline is not merely a direct consequence of aging but rather arises from other factors that sometimes are forgotten. We recommend further research addressing factors related to disability in local communities to design possible interventions for preventing functional loss in older adults.
